# One Side Ovarian Rejuvenation: A Quasi-Experimental Study of the Effect of the Autologous Platelet Rich Plasma in Poor Ovarian Responders in IVF

**DOI:** 10.4314/ejhs.v32i6.10

**Published:** 2022-11

**Authors:** Fatemeh Keikha, Saeideh Shahsavari, Yalda Salari, Nasibeh Roozbeh, Fedyeh Haghollahi, Masoumeh Dehghan Tarazjani, Maryamalsadat Razavi, Mamak Shariat, Maryam Bagheri

**Affiliations:** 1 Vali-E-Asr Reproductive Health Research Center, Family Health Research Institute, Tehran University of Medical Sciences, Tehran, Iran; 2 Fertility and Infertility Research Center, Hormozgan University of Medical Sciences, Bandar Abbas, Iran; 3 Medical Student, Brighton and Sussex Medical School, Brighton, United Kingdom; 4 Mother and Child Welfare Research Center, Hormozgan University of Medical Sciences, Bandar Abbas, Iran; 5 Assistant Professor, Department of Obstetrics & Gynecology, Ardebil University of Medical Sciences; 6 Maternal, Fetal & Neonatal Research Center, Family Health Research Institute, Tehran University of Medical Sciences, Tehran, Iran

**Keywords:** Platelet-Rich Plasma, Injections, Infertility, Female, Fertilization in Vitro

## Abstract

**Background:**

The poor ovarian response is the most important limiting factor in the success of in vitro fertilization (IVF). The aim of this study was to evaluate the outcome of intraovarian injection of autologous platelet-rich plasma (aPRP) on the oocyte number and IVF outcomes in poor ovarian responders (POR).

**Methods:**

This quasi-experimental study was performed from August 2021 to December 2021, in Vali-e-Asr Infertility Clinic affiliated with Tehran University of Medical Sciences, Tehran, Iran. There were 12 POR patients selected based on the criteria of Bologna group 4 who underwent two IVF cycles with similar antagonist regimens in a 70-day-interval. Immediately after the Oocytes Pick-Up (OPU), there was a 4cc of autologous PRP multifocal intramedullary injection done into their right ovaries in the first IVF cycle (case group). On the other hand, their left ovaries were considered as the control group. The patients underwent the second IVF cycle after 70 days.

**Results:**

Those who had undergone aPRP experienced a significant increase of the mean of antral follicular count (AFC) (from 1.91±0.79 to 2.50±0.90, p=0.043). There was a significant increase in the number of embryos from the right ovary (intervention group) compared to the left ovary (control group) after PRP, but there was no significant difference in the number of embryos in the right ovary before and after the intervention (from 0.25 ±0.45 to 1.08±0.79, p=0.705). There was no significant change in the number of oocytes, AMH, and FSH in the case and control groups before and after the intervention (p>0.05).

**Conclusion:**

According to the results of this study, it seems that in females with POR, intraovarian aPRP had no effect on the outcomes (embryos number, number of oocytes, FSH and AMH level), except for an increase in AFC.

## Introduction

In the current century, with an increase in the average maternal age (1), the number of couples seeking infertility treatment has increased (2). It is estimated that 10% of women may be at risk of unexplained reduced fecundity during the third decade of their lives. Over the course of time, ovaries experience a decrease in their follicular quantity (ovarian reserve) also known as poor ovarian reserve. These women may have a poor response to ovarian stimulation and experience early biological ovarian ageing (3–4).

The poor ovarian response is the most important limiting factor in the success of in vitro fertilization (IVF), which is observed in 9–24% of women undergoing assisted reproduction techniques (ARTs) (4). Due to increased age and reduction the ovarian reserve among these women, fertility and ovulation decreased (5).

There is no precise treatment to restore fertility in poor ovarian responders (POR). However, new treatments -such as ovarian rejuvenation using autologous platelet-Rich plasma (aPRP) are being tested and have shown promising results. Ovarian rejuvenation, which can be achieved by stem cell therapies, ovarian reactivation by tissue fragmentation, and attempting to generate oocytes in vitro. These interventions may be beneficial for infertile or subfertile women (6).

Utilization of aPRP in infertility practice has been suggested to beneficially affect follicle maturation in several ways and is a relatively new procedure offered to patients. The aPRP contains epidermal growth factor, vascular endothelial growth factor, basic fibroblast growth factor, platelet-derived growth factor, transforming growth factor, platelet-derived angiogenesis factor, and several interleukins. The results indicated that reproductive outcomes got better since conventional PRP was injected into ovarian tissue before IVF. Current leading hypothesis suggests that undefined growth factors released from platelets may induce the transformation of germline stem cells into primordial follicles, thus replenishing a diminished follicle pool. In addition, PRP would lead into intraovarian neovascularization. Moreover, due to the increase of cellular oxygenation or decrease in the concentration of intraovarian reactive oxygen species could modulate oocyte competence. PRP could increase intrastromal neovascularization as a result of which follicular perfusion would be improved and more importantly, embryo ploidy could be rescued. Rescuing embryo ploidy would happen through mRNA upregulation order by molecular signals resulting from PRP (7).

According to a recent study, aPRP may support ovarian function in poor responders (8). Recent clinical trials and animal studies have reported many beneficial effects of aPRP on infertility through its regenerative mechanisms. However, its clinical effect on infertility requires stronger evidence (9). Therefore, this study was conducted to evaluate the outcome of intraovarian injection of aPRP on the oocyte number and IVF outcomes in POR. We hypothesized that intraovarian injection of aPRP could improve the oocyte number and IVF outcomes in POR.

## Methods

This quasi-experimental study was conducted from August 2021 to December 2021 in the Vali-e-Asr Infertility Clinic affiliated with the Tehran University of Medical Sciences. Generally PRP will be offered to patients who had reached a point in their infertility treatment where the only remaining alternative treatment was perceived to be third-party oocyte donation. In this study, women with infertility for at least 12 months (infertility is defined as not being able to get pregnant after one year of unprotected sex), and poor response, based on the criteria of Bologna group 4 (10) to the antagonist stimulation protocols who were candidates for IVF were recruited.

Women with poor ovarian response according to group 4 of the Bologna criteria (10) who met at least two of the three criteria were included in the study. The criteria includes (a) advanced age (above 40 years) or any other risk factor for poor ovarian response (such as ovary surgery and ovarian endometrioma); (b) a previous poor response with no more than three oocytes retrieved using the conventional stimulation protocols; (c) abnormal ovarian reserve test results, including <7 total antral follicle counts (AFC) in both ovaries or <1.1ng/ml serum anti-mullerian hormone (AMH) (10), and the last analysis of their male partners contained at least 10 million sperms as well as at least 3% sperms with normal morphology. Only patients with a hemoglobin concentration of at least 11gr/dl and a platelet count of at least 150 × 109/L were entered into the study.

Women with a history of immunological or hematological diseases, chronic renal failure, respiratory tract infections, malignancy, endometriosis, submucosal myoma, Asherman syndrome, hypothyroidism, untreated hyperprolactinemia, other endocrine diseases, and any contraindication to pregnancy were excluded.

Other exclusion criteria were body mass index (BMI) above 30 or less than 18, systemic use of corticosteroids within two weeks before the procedure (Injected aPRP), any chromosomal or genetic abnormality, any pathology of the fallopian tubes (hydrosalpinx or pyosalpinx), and uterine anomaly.

None of the patients treated with supplements before the IVF cycles, and before all of the PRP procedures.

**Ovulation induction regimen**: Gonadotropin-releasing hormone (GnRH) Antagonist Protocol was used (Cinnal F ampule; 150 IU, Sc), Cinnajen Co., Iran) plus Human menopausal gonadotropin (HMG) (ampules; 150 IU, IM) (PD Homog Co, Iran) were administered daily from day three of the menstrual cycle. Follicle monitoring was performed five days later.

When the dominant follicles reached an approximate diameter of 14mm and were visualized by vaginal ultrasound (HS-2600, Honda Electronic Co., LTD, Japan, 12.5MHz), subcutaneous injections of GnRH antagonist (0.25mg Cetrotide (Merck Co, Germany)) were administered daily until the trigger day. When the dominant follicles reached a diameter of 16mm, the final stage of oocyte maturation was induced with an intramuscular injection of HCG (10000units) (PD Preg Co, Iran). The oocyte retrieval was performed 36 hours later. Following the ovarian puncture, aPRP injection into the right ovary was performed under general anesthesia by an infertility fellow as per the standard protocol (11–13). Women with any allergic reactions, lack of follicle growth suitable for ovarian puncture, and those who did not wish to continue the treatment were excluded at any stage. The obtained oocytes were fertilized by sperm in the laboratory, If there were any embryo, it would be frozen (embryo banking). During these two IVF cycles, no transfer of either fresh or frozen embryos were performed. After 70 days, the patients received the same first IVF treatment cycle of ovulation stimulation and an antagonist regimen. Due to the POR status of the patients, all resultant embryos were frozen and stored in the embryo bank.

**PRP procedure**: The aPRP protocol had two main steps: Step 1-Blood collection, centrifugation and aPRP aspiration. Step 2-Intra ovarian aPRP injection.

To perform aPRP, 10ml of blood was collected from the patient's peripheral vein, and the platelet count was checked. After two centrifugation cycles, the platelet count was rechecked. If the platelet count had increased by at least 4–5 times, four cc of aPRP was injected into the right ovary using a kit (RooyaGen Co; Iran). To prevent loss of growth factors, PRP extracted before clot formation. Immediately after the puncture, intraovarian injection was performed by a fixed infertility fellowship under anesthesia at four points via a 35cm 17G Cook Double Lumen Aspiration Needle using the guide of transvaginal ultrasound (HS-2600, Honda Electronic Co., LTD, Japan, 12.5 MHz). To eliminate possible interference of environmental factors on the results, the opposite ovaries (left) were assigned as the control group and did not receive the aPRP intervention.

**Hormone measurement**: Serum FSH (ACCU Bind ELISA kits, CA, USA) and AMH (ELISA method by pishtazteb.co.Iran kit) levels were measured on days 2–3 of menstrual cycle before the aPRP injection and about 70 days after the intervention with the same methods.The number of antral follicles were counted on the third day of menstruation, using two-dimensional transvaginal ultrasound by a fixed infertility fellowship (HS-2600, Honda Electronic Co., LTD, Japan, 12.5 MHz).

**Figure 1:**
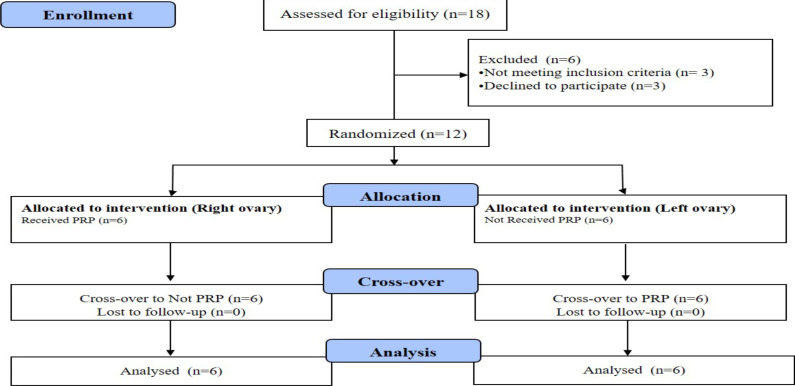
CONSORT flow diagram

**Sample size calculation**: Based on a previous study (14) in PRP-treated patients, the AMH level in the intervention and control groups were 1.01 and 0.58, with standard deviations of 0.4 and 0.3, respectively, assuming 95% confidence and statistical power 80%, the sample size in each group was estimated to be 11 women, which was assumed to be one-tenth of the probability of missing cases, we recruited 12 cases (Fig. 1).

**Data collection**: Demographic, clinical, and laboratory data were collected using a checklist. The information was extracted from the patients' records. The clinical and laboratory variables, including the total gonadotropin dose, FSH level, the number of dominant follicles (≥14 mm), mature oocytes (metaphase II), and available embryos (embryos meeting the standards of freezing), were recorded. Embryos were graded by an embryologist based on cell number and fragmentation.

**Outcomes**: The IVF results before and after aPRP injection were compared within the case and control group. The primary outcome included Antral Follicular Count (AFC), oocyte number, and the number and grade (according to the embryologist) of the embryos. On the other hand, the secondary outcome, included two hormonal profiles AMH level (ELISA method by Pishtaz teb Co. Iran kit) and FSH level (ACCU Bind ELISA kits, CA, USA) within the days 2–3 of menstrual cycle in the first and second IVF cycles.

**Statistical analysis**: Quantitative variables are described as mean±SD and percentage. The Chi-square test or Fisher's exact test was used to compare qualitative variables between the two groups. Since quantitative variables did not have a normal distribution, pre- and post-intervention values were compared using the Wilcoxon signed-rank test in each group and using the Mann-Whitney test between the two groups. The McNemar test was applied to compare qualitative variables after and before the intervention in each group (relative frequency of fetal quality). The analysis was done using the SPSS version 22 (SPSS Inc, USA). P-values < 0.05 were considered significant.

**Ethical considerations**: This study was approved by the institutional ethics committee of Tehran University of Medical Sciences (reference number: IR.TUMS.IKHC.REC.1400.191) and registered in the Iranian Registry of Clinical Trials (IRCT20210828052310N1). The study protocol was designed according to the ethical principles of the Declaration of Helsinki. All participants agreed to participate in the study, and written informed consent was obtained from all of them. The informed consent forms clearly stated that intraovarian PRP is an experimental therapeutic approach and it is not considered a clinical routine practice.

**Data sharing**: All relevant data and methodological detail pertaining to this study are available to any interested researchers upon reasonable request to corresponding author.

## Results

In total, 12 patients completed the study. The mean age and BMI of the subjects were 40.04±3.91 years and 22.59±9.76 respectively (Table 1).

**Table 1:** Patients' demographic and clinical characteristics

Variable	Frequency(Mean/%) n=12
Age (year)	40.40±3.91
BMI	22.59±9.76
Education	
High school education	7(58%)
University education	5(42%)
Job	
Housewife	8(67%)
employee	4(33%)
Type of infertility	
Primary	7(58%)
Secondary	5(42%)
Duration of infertility (year)	2.79±1.72
Previous IVF(n)	0.50±0.67

In the present study, aPRP except for an increase in AFC, had no effect on other outcomes. No significant difference was found in the endometrial thickness, the number of vials used to stimulate the ovaries, and the number of injection days before and after aPRP injection (p>0.05) (Table 2).

**Table 2:** Comparison of variables before and after intervention (aPRP)

Variable	Pre- intervention	Post-intervention	*P-value*
AMH	0.5±0.52	0.66±0.89	0.241*
FSH	18.22±16.99	16.06±11.41	0.224*
Endometrial thickness(mm)	2.40±0.51	2.33±0.49	0.317*
Number of FSH, HMG Ampule	3.83±0.57	4.00±0	0.317*
Number of injection days	12.41±2.74	12.50±1.93	0.730*

* Wilcoxon Ranks Test; Data presented as mean±SD. Wilcoxon Ranks Test; aPRP=autologous platelet-rich plasma; FSH=follicle-stimulating hormone; AMH=anti-Müllerian hormone; IVF= in vitro fertilization; AFC= antral follicle count.

No significant differences were detected in FSH and AMH levels before and seventy days after aPRP injection (Table 2). Following the intervention, more AFC was reported in the right ovary (intervention group) than in the left ovary (control group) (P=0.043) (Table 3).

**Table 3:** Comparison of variables between intervention and control groups.

Variable	Right ovary(n=12)	Left ovary (n=12)	P-value
**Number of Follicles**			
Pre-intervention	1.16±0.83	0.90±0.73	0.459*
Post- Intervention	1.58±0.90	0.40±0.84	0.674*
**Number of Oocytes**			
Pre-intervention	1.25±0. 96	1.00±0.70	0.203*
Post- Intervention	1.08±0. 99	1.00±0.70	0.418*
**AFC**			
Pre-intervention	1.91±0. 79	1.30±0/67	0. 080*
Post- Intervention	2.50±0. 90	1.60±0. 84	0.043*
**Number of embryos**			
Pre-intervention	0.25±0.45	0.30±0. 48	0.872*
Post- Intervention	1.08±0. 79	0.20±0. 63	0.014*
**Embryo Quality (pre -intervention)**			
A	1(20%)	1(30%)	0.322**
B	4(40%)	2(70%)	
AB	4(40%)	0	
**Embryo Quality (Post-intervention)**			
A	5(40%)	1(50%)	0.233**
B	3(20%)	0	
AB	5(40%)	1(50%)	

*Mann-Whitney Test t; **Chi-square TestData presented as mean±SD. * Mann-Whitney Test. * Data presented as n (%). **: Chi-square test, * aPRP: platelet-rich plasma, IVF: in vitro fertilization; AFC: antral follicle count

**Figure 2:**
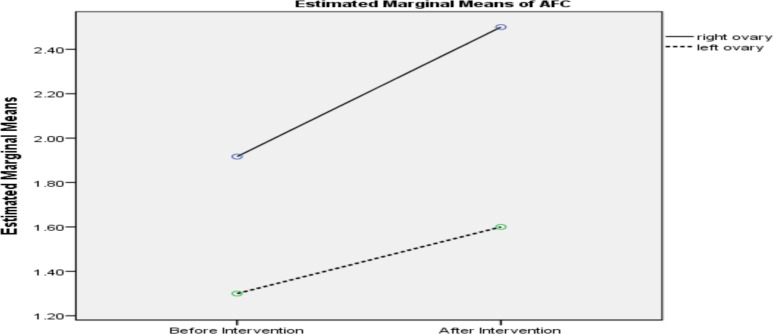
Comparison of AFC before and after intervention (aPRP)

The mean AFC increased from 1.91 ± 0.79 before the intervention to 2.50 ± 0.90 seventy days after the intervention (right ovary). The AFC significantly improved in the intervention group compared to the control group (p = 0.020 in the intervention group vs P=0.257 in the control group) (Table 4, Figure 2). The mean number of oocytes was 1.25 (0.96) and 1.08 (0.99) before and after aPRP injection. Additionally, after the intervention, the mean number of embryos in the right ovary was 1.08±0.79 and in the left ovary was 0.20 ±0.63. There was a significant increase in the number of embryos from the right ovary (intervention group) compared to the left ovary (control group) after PRP, but there was no significant difference in the number of embryos in the right ovary before and after the intervention (from 0.25 ±0.45 to 1.08±0.79, p=0.705) (Tables 3, 4). No significant difference was observed in the oocyte number between aPRP and control groups (p=0.608 vs p=0.317) (Table 4).

**Table 4:** Comparison of variables changes between intervention and control groups

Variable	Pre-intervention	Post-Intervention	P-value
Number of Follicles			
Right ovary	1.16±0.83	1.58±0.90	*0.096
Left Ovary	0.90±0.73	0.40±0.84	*0.046
Number of Oocytes			
Right ovary	1.25±0. 96	1.08±0.	*0.608
Left Ovary	1.00±0.70	1.00±0.70	*0.317
AFC			
Right ovary	1.91±0. 79	2.50±0. 90	*0.020
Left Ovary	1.30±0/67	1.60±0. 84	*0.257
Number of embryos			
Right ovary	0.25±0.45	1.08±0. 79	*0.705
Left Ovary	0.30±0. 48	0.20±0. 63	*0.660
Embryo Quality(Right ovary)			
A	1(20%)	5(40%)	**0.855
B	4(40%)	3(20%)	
AB	4(40%)	5(40%)	
Embryo Quality( left ovary )			
A	1(30%)	1(50%)	**0.905
B	2(70%)	0	
AB	0	1(50%)	

* Wilcoxon rank test

** Chi-square test

## Discussion

One of the biggest challenges in female infertility is the loss of ovarian reserve, which can occur at any age due to various reasons (11). In the present study, AFC increased after intra-ovarian injection of aPRP (into the right ovary) compared to the control group (left ovary). There was a significant increase in the number of embryos from the right ovary (intervention group) compared to the left ovary (control group) after PRP, but there was no significant difference in the number of embryos in the right ovary before and after the intervention. The number of oocytes, FSH and AMH level were not statistically significant change before and after intervention. In a study by Cremonesi et al., 5ml PRP (1 × 109ml) was injected into the right ovary of eight cows. APRP injection increased the number of follicles and increased the number of embryos (12). In a study by Farimany et al., 12 women suffering from poor ovarian reserve underwent ovarian stimulation and oocyte retrieval before and after injection of 2ml PRP (concentration not reported). The results showed an improvement in the indicators of ovarian reserve (reduced FSH level, increased AMH level, increased AFC) (13). Melo et al. conducted a non-randomized intervention study (PRP vs no injection). Their results showed an increase in AMH and AFC after treatment versus nonintervention, a decrease in the FSH level, an increase in the number of collected eggs, and a higher degree of resulting embryos (14). Given that in the present study, interpersonal factors are completely eliminated, it can justify different results from other studies with the present study. The sample size and eligibility criteria can affect the present study.

An increasing number of studies have shown that injecting PRP directly into the ovary can increase folliculogenesis and oocyte retrieval (15, 13). The difference in the outcome observed between the present study and other studies can be due to natural temporal and biological factors that may affect the process of ovulation stimulation (12–16). Different ovarian PRP methods, ovulation stimulation protocols and follow-up times, differences in the amount of material used in PRP, differences in the operator and ultrasound equipment may effect on results of studies (17). In our study, one operator and one device were used for all patients at all stages. Increased AFC in the aPRP treatment process may indicate effective treatment outcomes.

In a study by Navali et al. it has been indicated that single intra-ovarian of aPRP injection is associated with a significant elevation in the number of oocytes and embryos, as well as in the estradiol levels (18). Parvanov et al. in a study revealed that using autologous ovarian PRP in poor responders may be associated with a significant improvement in oocyte and embryo quality (19). Also, in another study showed that after intra-ovarian infusion of aPRP a significant improvement on the hormonal profile and the ovarian reserve status of patients occurred (20). However, the results of a study in Turkey showed that intra-ovarian injection of PRP do not increase live birth rates or clinical pregnancy rates in poor responder women or in those with premature ovarian insufficiency (21). According to the positive results of PRP therapy (12–16) and its reasonable cost, basic research on the effect of this treatment at a cellular level is needed to evaluate its effectiveness more comprehensively and to understand the mechanism of its effects.

This study have some limitations. One of the limitations of this study was the small sample size. In addition, we did not assess the safety and efficacy of this therapeutic method, its short-term and long-term side effects, and the pregnancy and live birth rates. Therefore, it is necessary to conduct cohort studies. The strength of this study was that the ovarian control group was on the opposite side and did not receive aPRP, resulting in more accurate results. In conclusion, according to the results of present study, in females with POR, intraovarian aPRP had no effect on the outcomes (embryos number, number of oocytes, FSH and AMH level), except for an increase in AFC.
